# Recommending Queries by Extracting Thematic Experiences from Complex Search Tasks

**DOI:** 10.3390/e20060459

**Published:** 2018-06-13

**Authors:** Yuli Zhao, Yin Zhang, Bin Zhang, Kening Gao, Pengfei Li

**Affiliations:** 1 Software College, Northeastern University, Shenyang 110004, China; 2 School of Computer Science and Engineering, Northeastern University, Shenyang 110004, China

**Keywords:** complex search, subtask identification, query recommendation, personalized PageRank

## Abstract

Since complex search tasks are usually divided into subtasks, providing subtask-oriented query recommendations is an effective way to support complex search tasks. Currently, most subtask-oriented query recommendation methods extract subtasks from plain form search logs consisting of only queries and clicks, providing limited clues to identify subtasks. Meanwhile, for several decades, the Computer Human Interface (CHI)/Human Computer Interaction (HCI) communities have been working on new complex search tools for the purpose of supporting rich user interactions beyond just queries and clicks, and thus providing rich form search logs with more clues for subtask identification. In this paper, we researched the provision of subtask-oriented query recommendations by extracting thematic experiences from the rich form search logs of complex search tasks logged in a proposed visual data structure. We introduce the tree structure of the visual data structure and propose a visual-based subtask identification method based on the visual data structure. We then introduce a personalized PageRank-based method to recommend queries by ranking nodes on the network from the identified subtasks. We evaluated the proposed methods in experiments consisting of informative and tentative search tasks.

## 1. Introduction

As the most important way to locate online information, search engines are being used to solve ever more complex search tasks such as writing survey papers and planning holiday trips. Although complex search tasks can occur under different situations [1,2] and take different forms [3], a general approach for solving search problems is to decompose them into subtasks and solve the subtasks one by one [4]. Thus, to help web searchers undertaking a complex search task, search engines need to identify the subtasks of the complex search task and help the searcher achieve each of the subtasks.

Query recommendation helps web searchers achieve a search task by recommending queries that better describe the search task [5]. The key idea of query recommendation is that, when a searcher sees a recommended query, the searcher can tell whether or not it is a better query to describe the current search task. To achieve this goal, query recommendation methods use different resources including search logs [4,6], social knowledge [7,8], and knowledge graphs [9,10] to provide recommendations. One important advantage of the search log-based query recommendation methods is that these methods can extract the subtasks of a search task by analyzing search logs, enabling these methods to provide subtask-oriented query recommendations. Such an advantage makes the search log-based query recommendation methods promising for supporting complex search tasks.

For search log-based query recommendation techniques, the key to success in supporting complex search tasks is to extract meaningful subtasks from the search logs [11]. Currently, subtasks are usually extracted from plain form search logs generated by commercial search engines consisting of queries, clicks, timestamps, and user/session IDs. Since almost all the commercial search engines are keyword-based one-shot search engines they can only generate plain form search logs, requiring the search log-based query recommendation methods to focus only on these plain form search logs. However, the Computer Human Interface (CHI)/Human Computer Interaction (HCI) communities have been developing new tools to support complex search tasks since the 1970s. By engaging rich user interactions, new designs such as SenseMap [12] and Apolo [13] can generate rich form search logs with much more information than is contained in plain search logs. Thus, new methods should be studied to extract subtasks from these rich form search logs generated by modern complex search tools for better subtask-oriented query recommendations.

In this paper, we report our results on providing subtask-oriented query recommendations by extracting subtasks from a proposed rich form of search log called Relative Chronological Source-tracking Tree (RCST). RCSTs are designed to capture the temporal, causal, and thematic experiences contained within a complex search task by organizing the queries and clicks of the search task as a relative chronological source-tracking tree. In an RCST, thematically related queries and clicks are organized into visually adjacent areas. Subtasks are then identified by splitting the RCST into visually isolated subtrees. Based on these subtasks, query recommendations are given by applying a personalized PageRank on the network generated by joining the subtrees.

The main contributions of this paper are summarized as follows:

- We formally introduce a visual data structure named Relative Chronological Source-tracking Tree (RCST) to capture the temporal, causal, and thematic experiences contained within complex search tasks.
- We seek to provide subtask-oriented query recommendations by leveraging the thematic experiences captured in RCSTs. We introduce a visual based method to identify subtasks from the RCSTs, and a personalized PageRank based method to find key queries from the query networks merged from the subtasks representing the thematic experiences from the crowd of searchers.
- The experimental results show that, compared with the methods extracting subtasks from plain form search logs, the proposed methods can identify subtasks and provide query recommendations with higher quality leveraging RCSTs as rich form search logs.

We organize the paper as follows: we introduce related works in Section 2. In Section 3, we describe the design of the relative chronological source-tracking tree (RCST) structure. In Section 4, we study the visual-based subtask identification method of RCSTs and propose the subtask-oriented query recommendation method based on the extracted subtasks. Then, in Section 5, we outline experiments used to verify the methods proposed in this paper. We finally conclude the work in Section 6.

## 2. Related Works

Although the problem of supporting simple search tasks has been well-studied, supporting complex search tasks is still an interesting question for investigation [14]. The CHI/HCI communities and the Information Retrieval (IR) communities are two important driving forces of this field of research.

Many complex search-supporting tools have been introduced in the past few years by the CHI/HCI communities. SearchBar [15] organizes queries and the corresponding clicks under user-entered topics to enable search task resumption and information re-finding. It works like a history log of queries and clicks organized in flat folders, which means that SearchBar is unable to organize queries in a hierarchal structure. sketchBrain [16] keeps track of queries and post-query click streams as graphs. A key feature of sketchBrain is that it logs the click streams after a user submits a query. Using this method, sketchBrain could log how a user finds information by tracking the navigation behavior of the user through web pages. However, sketchBrain organizes queries and click streams in a graphical manner, making it hard to review the whole search task when the search task becomes larger. IntentStreams [17] provides a horizontally scrollable workspace, with queries and search results listed vertically. In IntentStreams, related queries are located in a same vertical lane, with search results located on the bottom of the lane. A user could create a new lane by dragging and entering queries. Such a distinct structure makes it hard to determine the relationships between queries and clicks.

The design of the RCST is an extension of SearchBar and sketchBrain. The RCST extends SearchBar by allowing a searcher to organize queries on a topic in a tree structure, and extends sketchBrain by visually locating nodes in relative chronological order on a grid. These extensions make it possible to visually cluster nodes of an RCST into subtasks, potentially providing better query recommendations.

Query recommendation techniques, developed by IR communities, are effective in helping users discover new possible search directions in search tasks. A key step in recommending queries is revealing the relationships between queries. A typical approach is to analyze and build concept models from search logs, such as the concept hierarchy model-based method proposed in the research by Adeyanju et al. [18], and the query-flow graph shortcut path-based method proposed in the research by Anagnostopoulos et al. [19]. The click-through data in search logs contain implicit feedback, which could be used to gain deeper insights into concept relationships such as the query reformulation and click behavior simulation-based method proposed in the research by Zhu et al. [20], and the result preview click model-based method proposed in the research by Liu et al. [21].

Search log-based query recommendation methods generally limit query recommendations within the search systems, while some other studies have tried to incorporate data from different sources. Social knowledge is a widely used source of knowledge regarding concept relationships, such as the kernel-Princilpe-Component-Analysis-based method proposed in the research by Mao et al. [22], and the folksonomy-based methods proposed in the research by Otsuka et al. [7]. Social knowledge sources usually require knowledge analysis methods to extract knowledge from these sources, while formal knowledge sources like ontologies and knowledge graphs can provide an understanding of the relationships between concepts directly. As the largest single knowledge source Wikipedia is used in many query recommendation methods, such as the search direction discovery method proposed in the research by Yuvarani et al. [23]. The Linked Data project connects many formal knowledge sources, including Wikipedia, and thus has also been used for query recommendations [24]. A key problem in using formal knowledge sources for query recommendation is decisions regarding ranking of the different recommendations. To tackle this problem several methods have been proposed, such as the tag recommendation method-based concept ranking approach proposed in the research by Oliveira et al. [25]. However, unlike search log-based methods, these methods cannot extract subtask structures and thus are very limited in supporting subtasks for complex searches.

Although the aforementioned methods try to recommend queries to support complex search tasks, these methods still work in the so-called on-shot mode; that is, by optimizing query recommendations for the current query only. However, recently, more and more task/session-oriented search techniques have been introduced [26], such as the Partially observable Markov decision process-based search model proposed in the research by Grace et al. [27], and the item response theory-based retrieval model proposed in the research by Syed et al. [28]. Task/session-oriented query recommendation methods have also been proposed, such as the reinforcement learning-based method proposed in the research by Nogueira et al. [29], and the dynamic Bayesian network-based method proposed in the research by Guo et al. [30]. However, session/task based search techniques still face the problem of a lack of training data being available, as the distribution of queries usually follows a long tail distribution.

## 3. The RCST Visual Data Structure of the Search Log

In this section, we introduce our proposed visual data structure of the search log known as the RCST. The RCST is designed to capture the search experiences in complex search tasks. Like any other human experience, search experiences consist of four aspects: temporal experiences, cultural concepts of biography, causal experiences, and thematic experiences [31]. However, usually a search task will not last long enough for the cultural concept of biography to change. Thus, a complex search tool should help maintain temporal, causal, and thematic experiences of complex search tasks. However, the available complex search management tools fail to support all of these aspects of search experiences. We therefore designed the RCST structure to help web searchers maintain temporal, cultural, and thematic experiences in complex search tasks.

The RCST represents a complex search task as a tree. Queries and clicks of the search task are represented as tree nodes. The nodes are organized in a relative chronological source-tracking tree (RCST) structure. The RCST could be formally defined as follows:

*Node*. An RCST node is defined as N≔{G, C, T}, where G is the type of N, C is the content of N, and T is the timestamp of N.

*Node Type*. The type of RCST node N is defined as G∈{QUERY, CLICK}. If G is QUERY, then N is called a query node. If G is CLICK, then N is called a click node.

A query node is added to an RCST when a user submits a new query, while a click node is added when a user clicks a new search result.

*Node Content*. The content C of an RCST node N is a fragment of text. The content of N is determined by the type of N. If N is a query node, then C is the query keywords. If N is a click node, then C is the title of the clicked document. The RCST inherits all the definitions of a tree, such as the parent and sibling. The RCST also adds the following constraint definitions:

*Parent Node*. An RCST node A is the parent node of an RCST node B, if and only if there is an arc pointing from A to B. The parent node of an RCST node A always has a timestamp earlier than A.

*Brother Node*. An RCST node A is a brother node of an RCST node B, if and only if A and B share the same parent node, and the timestamp of A is earlier than the timestamp of B.

*Node Relation*. The relationship between two RCST nodes S and T (S≠T) is defined as R(S, T)∈{BROTHER, PARENT, OTHER}. If R(S, T) is BROTHER, then S is a brother node of T. If R(S, T) is PARENT, then S is the parent node of T. If R(S, T) is OTHER, then S is neither a brother node nor a parent node of T.

*Relative Chronological Source-Tracking Tree (RCST)*. The RCST of a search task is defined as T≔{NC, RC}, where ${\mathrm{NC}=\{n}_{1}{,\;n}_{2}{,\;\ldots ,\;n}_{p}\}$ is the set of nodes corresponding to the queries and clicks of the search task, and ${\mathrm{RC}=\{r}_{1}{,\;r}_{2}{,\;\ldots ,\;r}_{q}\}$ is the set of node relations between NC.

*Layout of the RCST*. An RCST is laid out on a grid using the Walker algorithm [32].

By definition, the following statements hold:

∀A, B, C∈NC, if R(A, B) and R(B, C) ∈ {BROTHER, PARENT}, then A has a timestamp earlier than C.

(1) ∀A, B ∈ NC, R(A, B) ≠ R(B, A) if R(A, B) or R(B, A) ∈{BROTHER, PARENT}.
(2) ∀A, B, C∈NC, if R(A, B) = R(B, C) = PARENT, then R(A, C) = OTHER.
(3) ∀A, B, C∈NC, if R(A, B) = R(B, C) = BROTHER, then R(A, C) = BROTHER.

**Figure 1:**
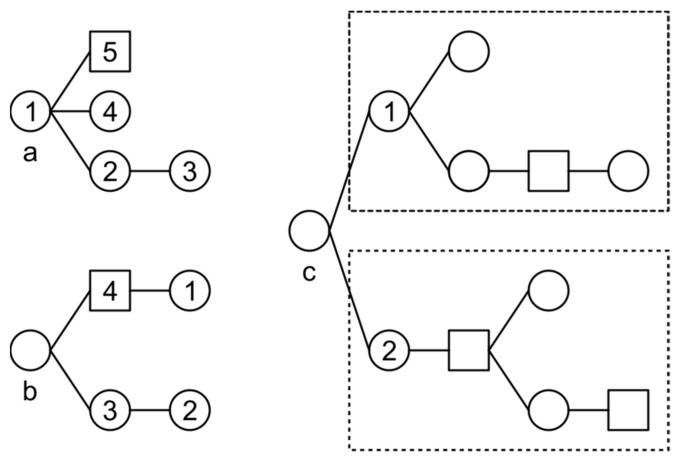
Examples of identifying time orders, provenances, and subtasks from an RCST. (**a**) The time order is 1-5-4-2-3; (**b**) Query node 1 comes from the content of the document of click node 4; (**c**) As the size of subtrees rooted as node 1 and 2 increases, the visual distance between nodes 1 and 2 increases, making it easy to separate two subtasks.

Representing a search task as an RCST helps a searcher identify the temporal, causal, and thematic experiences of the search task, as shown in Figure 1. To determine the time order of ∀A, B∈NC, if R(A, B) ∈ {BROTHER, PARENT}, or ∃R = {R(A, C_1_), R(C_1_, C_2_), …, R(C_n−1_, C_n_), R(C_n_, B)} so that ∀r∈R, r ∈ {BROTHER, PARENT}, then A has a timestamp earlier than B. To determine the provenance of A ∈ NC, if A is a click node, then ∃R(B, A) = PARENT, B ∈ NC so that A is clicked in the search engine result page (SERP) of B. If A is a query node, then if ∃R(B, A) = PARENT, B ∈ NC and B is a click node, then A comes from the content of the document of B. If B is a query node, then A is a post query of B, and may be a refinement of B, or comes from the user’s knowledge or the task requirements.

To determine the subtask structure of a complex search task, for ∀A∈NC there is no more than one B ∈ NC so that R(B, A) = PARENT, but there could be a set of nodes **NCS** = {A, S_1_, S_2_, …, S_n_} ⊆ **NC** so that ∀S_i_ ∈ NCS − {A}, R(A, S_i_) = PARENT. Given such an **NCS**, as the size of **NCS** increases, the visual distance between A and the siblings of A increases as well since the RCST is laid out on a grid using the Walker algorithm. Such visual separations intuitively separate an RCST into several components, helping the web searcher to identify subtasks from the RCST.

## 4. Recommending Subtask-Oriented Queries Using RCSTs

In this section, we identify subtasks from RCSTs, and recommend queries based on the subtasks identified. We first identify subtasks from the RCST in a visual based manner. It is then given a user query, and subtasks containing the query are merged into a network. We then calculate a ranking score to determine which recommendations should be provided.

### 4.1. Visual-Based Subtask Identification from RCSTs

As discussed in Section 3, when a search task is organized as an RCST, the subtask structure intuitively separates the RCST into several visual components. This section describes an algorithm to leverage such visual separations to extract subtasks from the RCST, as shown as Algorithm 1.

**Algorithm 1.** Subtask Extraction Algorithm for RCST**Input**: The RCST of a search task, distance d**Output**: SubtaskSet = {NodeSet_1_, NodeSet_2_, …} extracted from the RCST
**1.** 
**Begin**
2.SubtaskSet = {} // A set of node set, each node set corresponds to a subtask3.**For each** RcstNode in RCST4.WalkerLocate(RcstNode, d)5.SetRange(RcstNode, 2d)6.SubtaskSet << {RcstNode}7.
**End For**
8.**For each** set pair (S1, S2) in SubtaskSet9.**If** RangeOverlapping(S1, S2)10.**If** exists N in S1, R(N, Root(S2)) == PARENT || BROTHER11.SetMerge(S1, S2)12.
**End if**
13.
**End if**
14.**End For** if no set pair is merged15.**Export** SubtaskSet**16.** 
**End**



The RCST of a search task and distance d are fed to Algorithm 1 as inputs, and the algorithm is expected to return a set of node sets SubtaskSet with each node set representing a subtask extracted from the RCST.

**Figure 2:**
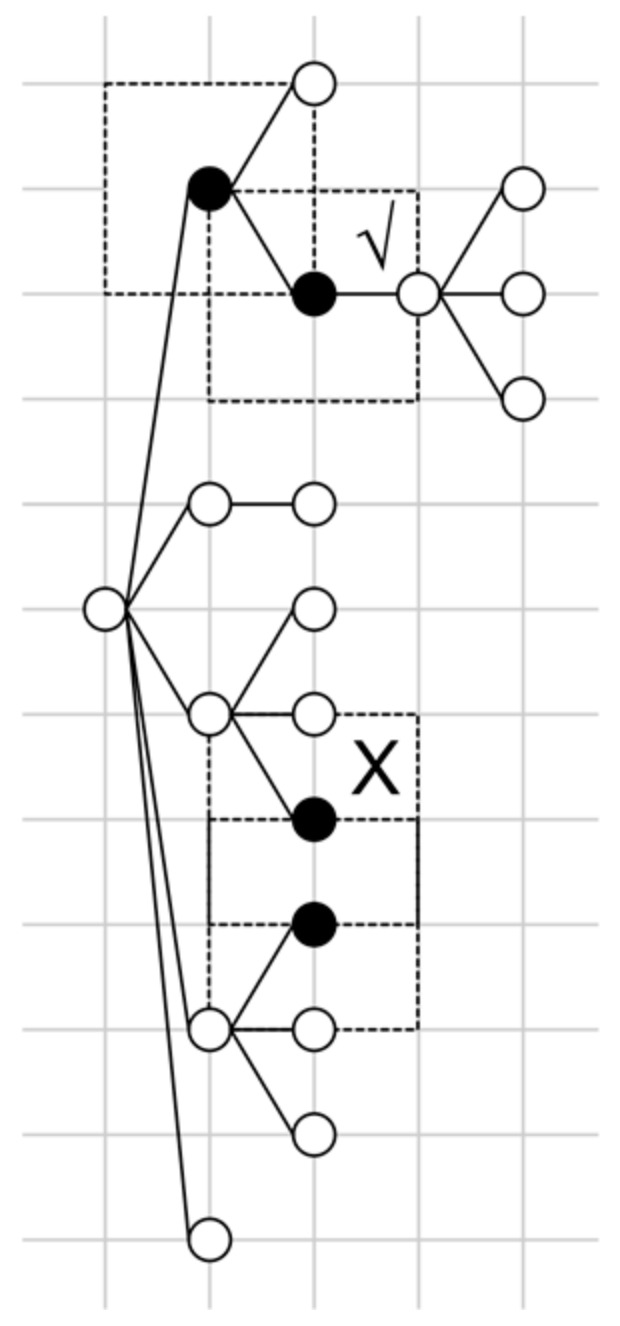
Merging subtasks in an RCST. The upper two black nodes are merged. The lower two black nodes, although they have overlapping ranges, cannot be merged since the relationship between the two nodes in both directions is OTHER.

As the first step, SubtaskSet is set to empty. Then, the nodes of the RCST are laid out on a grid using the Walker algorithm [32], as shown in Figure 2. The spacing of the grid is set to d. For node A, the parent node of A is located to the left of A. The brother nodes of A are located above A. Then, for each node, a 2d by 2d rectangle range is drawn centered on the node.

A subtask is a set of nodes in the RCST. At the start of Algorithm 1, each node is treated as a subtask and is added to SubtaskSet. Then, for each pair of subtasks (S1, S2) in SubtaskSet, if the rectangle ranges of S1 and S2 overlap, and there exists a node N in S1 so that N is the parent or brother of the root node of S2, then S1 and S2 are merged as a new subtask. The algorithm ends when no pair can be merged.

### 4.2. Subtask Identification from Node Chains

**Figure 3:**
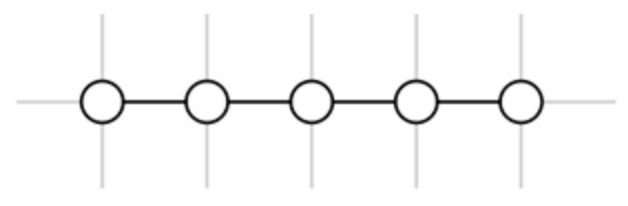
An example of a node chain.

A special case in an RCST is a set of nodes **N** = {n_1_, n_2_, …, n_k_} where ∀n∈N; there is no n′ so that R(n, n′) = BROTHER or R(n′, n) = BROTHER. Such a set of nodes looks like a chain when they are laid out on a grid. We refer to such a set of nodes as a node chain, as shown in Figure 3.

A node chain is a set of nodes with no brothers, corresponding to a series of queries and clicks with no branches. Node chains occur when a searcher is:

(1) Exploring different possible search directions. The searcher does not know which search direction to follow, and is trying a series of different possible directions. These possible directions may be related, but do not form a clear structure to fit into a tree structure.
(2) Exploiting a specific topic. The searcher has found a possible search direction, and is digging into that topic.

The visual-based subtask identification algorithm introduced in Section 4.1 relies on brother relations to visually identify subtasks from an RCST. Since node chains contain no brother relations, it is impossible for the algorithm to identify subtasks from node chains. To overcome this shortcoming, we designed a rule-based method to identify subtasks from node chains. The rules were designed for the exploring/exploiting situations as follows:

(1) When a searcher is exploring different possible search directions, each possible direction in the node chain is treated as a separate subtask.
(2) When a searcher is exploiting a specific topic, the whole node chain is considered a single subtask.

We adapt the win-win search framework proposed in the research by Luo et al. [33] to identify exploration and exploitation queries from node chains. The win-win search framework identifies query states on two dimensions; the relevant dimension and the exploration dimension. However, in our setup, we only considered the exploration dimension. Thus, the observation function O (s_j_, a_t_, ω_t_) [33] is calculated only by:

O (s_t_ = Exploitation, a_u_ = Δq_t_, Σ_se_ = D_t−1_, ω_t_ = Exploitation) ∝P (s_t_ = Exploitation|ω_t_ = Exploitation) × P (ωt = Exploitation| = Δq_t_, D_t−1_) (1)

O (s_t_ = Exploration, a_u_ = Δq_t_, Σ_se_ = D_t−1_, ω_t_ = Exploration) ∝P (s_t_ = Exploration|ω_t_ = Exploration) × P (ωt = Exploration| = Δq_t_, D_t−1_) (2)

In Equations (1) and (2), Δq_t_ represents the query changes between the current query q_t_ and the previous query q_t−1_, and D_t−1_ represents the document set returned by the search engine according to q_t−1_. The idea behind Equations (1) and (2) is that [33], if added query terms appear in D_t−1_, then the user may stay at the same topic for exploitation. If the added terms do not appear in D_t−1_, then the user may move to the next topic for exploration. Meanwhile, if there are deleted terms, then the user may go to a broader topic to explore. If there are no added and deleted terms, the user may fall into exploitation. More detailed outcomes about exploration and exploitation in search tasks can be found in [34,35,36].

The state of the first query in a node chain, (s_0_) is set to exploration. After labeling the state of all the queries in a node chain, subtasks are identified according to the aforementioned rules.

### 4.3. Merging Subtasks into Networks

A search subtask extracted from an RCST contains the thematic experiences of a searcher to complete the subtask. However, it is generally impossible to determine which user experience is the best experience to complete a search subtask. Instead, to provide subtask-oriented query recommendations, experiences from the crowd of searchers should be considered. To achieve this goal for a given query, we first merge the subtasks related to the query into a network. The algorithm used to merge subtasks is Algorithm 2, shown below.

**Algorithm 2.** Subtask Merging Algorithm.**Input**: Query Q, Subtask set SubtaskSet**Output**: Network for query Q
**1.** 
**Begin**
2.QSubtaskSet = {}3.**For each** Subtask in SubtaskSet4.**For each** query q in Subtask5.**If** Similar(Q, q)6.QSubtaskSet << Subtask7.
**End If**
8.
**End For**
9.
**End For**
10.Graph.VertexSet = {}11.Graph.ArcSet = {}12.**For each** Subtask in QSubtaskSet13.Graph.VertexSet = SetUnion(Graph.VertexSet, Subtask.QuerySet)14.**For each** pair (S1, S2) in Subtask15.**If** R(S1, S2) == PARENT || exists N in Subtask.ClickSet,16.R(S1, N) == R(N, S2) == PARENT17.Graph.ArcSet << (S1, S2)18.(S1, S2).Weight = |**N**|, ∀N∈N, R(S1, N) == R(N, S2) == PARENT19.(S1, S2).Weight++ **If** R(S1, S2) == PARENT20.
**End If**
21.
**End**
22.
**End For**
**23.** 
**End**



In Algorithm 2, given query Q and subtask set SubtaskSet, subtasks containing query Q are selected as set QSubtaskSet. The selection is made by checking the similarity between query Q and each of the query in a subtask according to a similarity function Similar (). In real world applications, the similarity function could be implemented by simple keyword matching, by a porter stemmer, or by combined similarity functions like the one introduced in the research by Mehrotra et al. [11].

**Figure 4:**
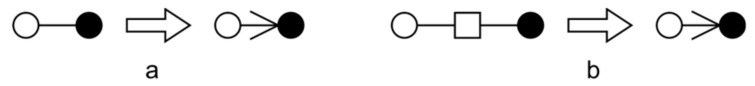
Demonstrations of creating arcs for the merged complex network of subtasks. (**a**) An arc for directly connected query nodes; (**b**) An arc for query nodes connected by a click node.

The set of subtasks containing query Q is then merged into a complex network referred as Graph of query nodes. The vertex set of Graph contains all the query nodes of the subtask set by merging the query set of each of the subtasks in QSubtaskSet into the vertex set of Graph. For arcs of Graph, if there exists an arc pointing from query node S1 to query node S2 in the RCST of a subtask, then an arc pointing from S1 to S2 is created in the result complex network, as shown in Figure 4a. If there exists a direct path of two hops connecting query node S1 and query node S2 with a click node N, an arc connecting S1 and S2 is also created in the resulting complex network, as shown in Figure 4b. After all the arcs are created duplicated arcs connecting any pair of nodes are merged, with the weight of the merged arc set to the number of duplications.

### 4.4. Recommending Queries from the Merged Query Network

For a query Q, based on the network merged from subtasks containing Q, we now recommend queries for Q. Subtasks identified from RCSTs contain thematic experiences showing how to complete search subtasks. When recommending queries to a searcher, instead of providing only a list of query recommendations, we also want to reveal the thematic experiences relating to how to complete the corresponding search task. To achieve this goal, we not only find queries to recommend for a given query Q, but also find search paths that lead to these recommendations. Thus, the job of recommending queries from a merged query network could be divided into two parts: (1) extracting query recommendations from the query network, and (2) find optimized search paths leading to the recommendations.

#### 4.4.1. Extracting Query Recommendations from Query Networks

Given query Q and the corresponding query network N, we seek to find query recommendations for Q from N. As described in Section 4.3, the query network N is created by merging subtasks related to query Q. Thus, we could say that the query network N contains the thematic experiences of the crowd of searchers who have achieved the subtasks. We therefore try to find key queries from query network N that are mostly used by the crowd to achieve the subtasks.

We assume a random searcher who has the crowd thematic experiences, i.e., query network N, in his mind. The random searcher always starts from query Q, and randomly picks a query q from the neighbors of Q in query network N. The probability of choosing query q is proportional to the weight of the arc connecting query Q and query q. After query q is chosen, the random searcher starts the same process again from q, and randomly chooses a new query q′ from the neighbors of q. When choosing a neighbor, the random searcher may decide to restart at query Q by probability 1 − d. Such a random search process simulates how a searcher achieves subtasks based on the crowd thematic experiences. The rank of each query node is then determined by the probability the random searcher landing on the node.

The aforementioned random search process corresponds to a personalized PageRank on network N, personalized by query Q. The rank of query node i is therefore calculated as:

(3) $$\begin{array}{c} \mathrm{PR}\left(i\right)=\left(1-d\right)r_{i}+d\underset{j\in \mathrm{in}(i)}{\sum }\frac{\mathrm{PR}(j)\times \mathrm{out}(j,i)}{\mathrm{out}(j)}\\ r_{i}=\{\begin{array}{cc} 1 & i=Q\\ 0 & i\neq Q \end{array} \end{array}$$

where PR(i) is the rank of query node i, 1 − d is the probability that the random walk restarts from query Q, in(i) is the set of query nodes with arcs pointing to query node i, out(j,i) is the weight of the arc pointing from query node j to query node i, and out(j) is the sum of all the weights of the arcs pointing from query node j. The random walk is personalized by query Q. When the random walk restarts with a probability of 1 − d, it will restart at query Q instead of a randomly chosen node. After the PR scores are calculated for each node, top-K nodes are selected as recommendations.

#### 4.4.2. Finding Optimized Search Paths for Recommendations

After extracting recommendations from query network N with respect to query Q, we then find optimized search paths from Q to each of the recommendations. For a recommendation K, we try to find a path which starts from Q leading to K with maximum probability. The probability of a path (Q, $q_{1}$, $q_{2}$, …, $q_{n}$, K) is defined as:

(4) $$P\left(Q,q_{1},q_{2},\ldots ,q_{n},K\right)=P\left(Q,q_{1}\right)P\left(q_{1},q_{2}\right)\ldots P(q_{n},K)$$

where:

(5) $$P\left(i,j\right)=\frac{\mathrm{out}(i,j)}{\mathrm{out}(i)}$$

We then find the optimized set of ($q_{1}$, $q_{2}$, …, $q_{n}$) that maximizes the path probability for K given Q.

## 5. Experimental Evaluations

In this section, we evaluate the methods proposed in this paper. We first evaluate the visual-based subtask identification method. We then evaluate the query recommendation method based on the thematic experiences extracted from the RCST.

### 5.1. Evaluations of the Visual-Based Subtask Identification Method

We first evaluate the visual based subtask identification method by comparing the method with a similarity-based subtask identification method. We designed four search tasks—two informative search tasks and two tentative search tasks—and recruited 24 volunteers to complete the search tasks. We then apply the proposed and comparison methods to identify subtasks from the search tasks. The results are evaluated according to a set of manually identified subtasks.

#### 5.1.1. Experimental Setup

The proposed visual-based subtask identification method leverages the RCST structures of search tasks. The key idea of the proposed method is based on the observation that searchers tend to organize queries and clicks of a subtask together, forming visual clusters that separate the subtask from other subtasks. In traditional query logs this visual information, as well as the RCST structure, is not available. Thus, we compared the proposed method to a traditional subtask identification method.

As the proposed method only extracts flat subtasks from RCSTs, we considered a modified version of the subtask identification method proposed in the research by Mehrotra et al. [11]. We considered four features to measure the degree of similarity between two queries: (1) TF-IDF [37]-based cosine similarity between query texts; (2) user similarity showing if two queries are issued by the same user; (3) TF-IDF-based cosine similarity between document contents; and (4) text embedding-based cosine similarity between query texts. The four features are normalized and summed to measure the degree of similarity between two queries. Based on such a similarity measure, K-Means [38] clustering is used to identify subtasks from query logs. This multiple features K-Means approach is referred to as MF K-Means. We also adapted the methods proposed in the research by Wang et al. [39] and Li et al. [40], which are referred as Bestlink-SVM and LDA-Hawkes, respectively.

The subtask identification results of the four methods were evaluated according to a set of manually identified subtasks. The average precision values of the identification results were used to evaluate the two methods. Based on the manual subtask identification result A, by checking if any two queries belonged to the same cluster in A and in the result of a subtask identification method B, four possible situations could occur:

(1) DS: the two queries are in different clusters in A but in the same cluster in B.
(2) DD: the two queries are in different clusters both in A and B.
(3) SS: the two queries are in the same cluster both in A and B.
(4) SD: the two queries are in the same cluster in A but in different clusters in B.

By counting the number of DS, DD, SS, and SD situations, we were able to calculate the positive precision AP, negative precision AN, and average precision AA as:

(6) $$\begin{array}{c} \mathrm{AP}=\mathrm{SS}/(\mathrm{SS}+\mathrm{SD})\\ \mathrm{AN}=\mathrm{DD}/(\mathrm{DD}+\mathrm{DS})\\ \mathrm{AA}=(\mathrm{AP}+\mathrm{AN})/2 \end{array}$$

We designed four search tasks. The first two search tasks were designed to be informative and were completed in Chinese, which was the native language of the volunteers. The first two search tasks were on topics that the volunteers had heard of but were not familiar with, and thus exploration of the topic was required. The last two search tasks were designed to be tentative and were completed in English, which was the second language of the volunteers. The volunteers were asked to read the abstracts of two papers written in English without knowing the purpose of doing so. Then, two days later, the volunteers were asked to find a list of papers related to the abstracts. The volunteers were asked to write reports to summarize their findings after each search task. The four search tasks are listed below:

(1) Write a report about drugs used in chemotherapy. Introduce common drugs used in chemotherapy, including their pharmacological actions, indications, administrations, and side effects. Introduce treatment strategies and common combinations of chemotherapy regiments. Do not focus only on drugs that beat cancer. Consider also drugs that decrease the toxic effects of other drugs.
(2) Write a report about fine particles (PM_2.5_) in China. Introduce the concept and sources of PM_2.5_. Introduce how and why PM_2.5_ affects human health. Introduce the top affected cities by PM_2.5_ in China. Analyze the causes of PM_2.5_ in the top affected cities. Introduce methods as well as the corresponding mechanisms and feasibilities to reduce PM_2.5_. Analyze both the positive and the negative effects of the introduced methods.
(3) Abstract of S. Shunmuga Krishnan, Ramesh K. Sitaraman. Video Stream Quality Impacts Viewer Behavior: Inferring Causality Using Quasi-Experimental Designs. *IEEE/ACM Trans. Netw*. **2013** *21*(6), 2001–2014.
(4) Abstract of Hanqiang Cheng, Yu-Li Liang, Xinyu Xing, Xue Liu, Richard Han, Qin Lv, Shivakant Mishra: Efficient misbehaving user detection in online video chat services. *WSDM* **2012**, 23–32.

Twenty-four graduate students majoring in computer science were recruited as volunteers to perform the search tasks, for a total of 96 complex search tasks. Based on the reports, the following subtasks were extracted for each search task:

(1) Introductions, drugs, drug mechanisms, how to use drugs, indications, side effects, treatments, combinations of drugs, ancillary drugs, miscellaneous.
(2) Introductions, concepts, sources, damages and reasons, PM_2.5_ in China, most affected cities and reasons, measures against PM_2.5_, feasibilities.
(3) Web video service, web video charge, video category, user behavior, video quality, web video safety, web video revisit.
(4) Video chat content, online chat misbehavior, online chat algorithms.

When manually identifying subtasks, we adapted a positive strategy to manually cluster queries: when a query could be put in multiple clusters, it was added to each of the possible clusters.

#### 5.1.2. Experimental Results

We first introduce some basic statistics about the experimental results. On average, a volunteer made 65.8 queries and 55.3 clicks (0.84 click per query) in an informative search task, and 48.6 queries and 30.3 clicks (0.62 click per query) in a tentative search task. The informative search tasks caused 35.5% more click per query than the tentative search tasks. We believe this was because that in informative search tasks, a volunteer needed to click more to collect information, while in tentative search tasks, a volunteer needed to query more to look for possible search direction.

Volunteers could reorganize RCSTs by dragging a node, as well as the subtree rooted at the node, apart from its parent and dropping the node on another node N to set N as the parent of the node. On average, a volunteer made 5.25 drag-n-drops in an informative search task, and 1.83 drag-n-drops in a tentative search task. We think this was because that tentative search tasks trended to show more linear structures compared with informative search tasks. It was harder to organize tentative search tasks as trees, so less drag-n-drops were observed in tentative tasks. Meanwhile, informative search tasks usually had clearer subtask structures than tentative search tasks, and the volunteers could reflect these structures by drag-n-dropping. However, although informative search tasks led to more drag-n-drops, we think the expense was still low considering the number of queries and clicks made.

The proposed and the compared subtask identification methods were applied to the 96 complex search tasks. The AP, AN, and AA metrics were calculated for informative and tentative search tasks separately. The results are shown in Table 1.

From Table 1 we can see that the proposed method outperforms the compared methods in both informative search tasks and tentative search tasks, while providing an intuitive visual structure of subtasks. The advantages are more obvious for informative search tasks than tentative search tasks. We think this is due to the fact that the compared methods are in essence similarity-based. Although queries of a subtask are usually similar to each other, there are also many exceptions: similar queries may belong to different subtasks, while different queries may belong to the same subtask. These exceptions limit the performance of similarity-based methods. Meanwhile, since users tend to organize queries of the same subtask in the same region to ease the management of search tasks, the proposed method performs better in identifying subtasks.

In tentative search tasks, the advantages of the proposed method are not as obvious as in informative search tasks. This is because the key aim of a tentative search task is to find a set of keywords leading to a specific yet ambiguous target. In tentative search tasks, a user usually forms a linear search path. A subtask in a tentative search task is usually a set of similar queries on the same topic, forming a node chain in the corresponding RCST. The similarity-based text clustering methods (the compared methods) can perform well with these data. On the other hand, since node chains cannot be separated by the visual-based algorithm proposed in Algorithm 1, their performance is limited compared with that of informative search tasks.

### 5.2. Evaluations of the Proposed Query Recommendation Method

To evaluate the proposed query recommendation method, we used the dataset from Section 5.1.1 to train the recommendation model. As a comparison, we also applied the same recommendation method proposed in Section 4.4 to the compared methods used in Section 5.1.1. Another six students were recruited to perform the same tasks as introduced in Section 5.1.1. When a volunteer submits a query, each method is allowed to provide three recommendations to the volunteer. The volunteers were asked to evaluate whether each recommendation was useful. Volunteers were also asked to submit new queries if they had new search directions that were not covered by the recommendations. We then adopted traditional precision and recall as evaluation measures. The evaluations were undertaken for informative and tentative search tasks separately. The results are shown in Table 2.

From Table 2 we can see that the proposed thematic experience-based query recommendation method achieved higher precision and recall values on both types of search tasks. We believe this advantage is due to the fact that the proposed method could identify subtasks with higher precision, as shown in Section 5.1.2.

### 5.3. Complexity of Algorithms

We analyzed the complexity of the algorithms used in the proposed query recommendation method.

For Algorithm 1, the Walker algorithm operates in time O(N). For a node located on a grid, the overlap checking can be achieved by checking the eight neighbors located within the 2d by 2d rectangle range. Thus, the time complexity of the overlap checking, as well as Algorithm 1, is O(N). The space required for Algorithm 1 is also O(N).

For the subtask identification algorithm for node chains, after the models are trained the time required to calculate the observation function is O(M) where M is the number of clicks for a query, and the overall time required by the algorithm is O(M × N) where N is the number of queries in a node chain. The space required by the algorithm is O(N) since each query requires two spaces to store the value of the observation function and the state of exploration/exploitation.

For Algorithm 2, the time required to build QSubtaskSet is O(N) where N is the number of subtasks in SubtaskSet. To merge the subtasks in QSubtaskSet, we need to traverse all the node pairs in a subtask, leading to a time complexity of O(M^2^) where M is the number of nodes in the subtask. Then, to merge all the subtasks in QSubtaskSet, the time required is O(NM^2^). The space required by the algorithm is the sum of all the spaces taken by each subtask in QSubtaskSet.

The time complexity of PageRank is O(N + M) where N is the number of nodes and M is the number of arcs. The space required by PageRank is O(N).

## 6. Conclusions

In this paper, we proposed a query recommendation method based on the thematic experiences extracted from rich form search logs captured in a proposed visual data structure of complex search tasks, called an RCST. We designed a visual-based subtask identification method to extract thematic experiences from RCSTs. The extracted subtasks are then combined into a network, and query recommendations are provided by applying personalized PageRank on the network. The visual-based subtask identification method and the query recommendation method were evaluated for both informative and tentative search tasks. The results showed that the proposed method is effective in supporting complex search tasks.

## Figures and Tables

**Table 1:** Subtask identification results for informative and tentative search tasks.

Method	Informative Search Task			Tentative Search Task		
	AP	AN	AA	AP	AN	AA
Proposed	0.508	0.794	0.651	0.553	0.589	0.571
MF K-Means	0.371	0.786	0.576	0.536	0.563	0.550
Bestlink-SVM	0.366	0.772	0.569	0.533	0.552	0.543
LDA-Hawkes	0.360	0.764	0.562	0.524	0.542	0.533

**Table 2:** Evaluation results of the query recommendations.

Task Type	Measures	Proposed	MF K-Means	Bestlink-SVM	LDA-Hawkes
Informative	Precision	0.552	0.483	0.474	0.478
	Recall	0.713	0.624	0.612	0.617
Tentative	Precision	0.483	0.469	0.464	0.453
	Recall	0.642	0.624	0.616	0.601
